# Hearts apart: sex differences in cardiac remodeling in health and disease

**DOI:** 10.1172/JCI180074

**Published:** 2024-07-01

**Authors:** Thomas G. Martin, Leslie A. Leinwand

**Affiliations:** 1 Department of Molecular, Cellular, and Developmental Biology and; 2 BioFrontiers Institute, University of Colorado Boulder, Boulder, Colorado, USA.

## Abstract

Biological sex is an important modifier of physiology and influences pathobiology in many diseases. While heart disease is the number one cause of death worldwide in both men and women, sex differences exist at the organ and cellular scales, affecting clinical presentation, diagnosis, and treatment. In this Review, we highlight baseline sex differences in cardiac structure, function, and cellular signaling and discuss the contribution of sex hormones and chromosomes to these characteristics. The heart is a remarkably plastic organ and rapidly responds to physiological and pathological cues by modifying form and function. The nature and extent of cardiac remodeling in response to these stimuli are often dependent on biological sex. We discuss organ- and molecular-level sex differences in adaptive physiological remodeling and pathological cardiac remodeling from pressure and volume overload, ischemia, and genetic heart disease. Finally, we offer a perspective on key future directions for research into cardiac sex differences.

## Introduction

Biological sex influences nearly every aspect of human physiology and has important implications for many diseases. Heart disease is the leading cause of death for both men and women, but sexual dimorphisms exist in disease development and clinical outcomes (1). Male and female cells and animals are not identical; however, prior to the mandated inclusion of women in NIH-funded studies in 1993, clinical trials predominantly included men (2). This contributed to the development of therapies and recommended dosages that often favor positive outcomes in men (3, 4). In animal models, females were infrequently studied due to the incorrect assumption that males were less variable. It has since been demonstrated that female mice introduce variability equal to or less than that of males, irrespective of estrous cycle stage (5, 6). Federal funding agencies now require studying both the male and female sexes when possible, and research into sex differences in cardiac biology has become increasingly prevalent over the last two decades.

The heart is a dynamic organ and constantly interprets and responds to internal and external cues. Examples include cardiac adaptation occurring with endurance exercise training, where increased circulatory volume triggers reversible cardiac growth that is associated with improved function (7). Remodeling is also common in pathological settings, including aortic stenosis (AS) and aortic regurgitation, where increased left ventricular (LV) pressure and volume, respectively, trigger maladaptive growth that causes dysfunction (7). Sex differences are well documented in both physiological and pathological cardiac remodeling. In pathological settings, premenopausal women demonstrate protection against adverse remodeling, maintain better cardiac function, and have reduced mortality compared with men. This is often attributed to distinct cell signaling elicited by female and male sex hormones, although additional factors also influence cardioprotection in females.

In this Review, we first define baseline structural, functional, and molecular differences between male and female hearts to provide context. Additionally, we highlight factors influencing sex-biased cellular signaling, including circulating hormones and sex chromosomes. We then discuss sex differences in physiological and pathological remodeling. Sex differences in genetic heart disease are also prevalent, and we review the literature on these conditions. Finally, we offer our perspective on future directions for research into cardiac sex differences. In this study, we discuss only the role of chromosomal sex, which is a biological construct. The influence of gender — a nonbinary social construction — on cardiovascular disease risk and outcomes has been reviewed previously (2).

## Baseline differences between male and female hearts

Cardiac sex differences begin in utero, are enhanced during puberty, and continue throughout adulthood. The purpose of this section is to highlight baseline differences between male and female hearts and thus establish context to inform the following discussion of sex biases in cardiac remodeling.

### Sex differences in cardiac structure and function.

Cardiac sex differences begin in utero, where male fetuses experience increased LV preload (stretch induced by ventricular blood volume at the end of diastole) and reduced afterload (impedance against which the LV works to eject blood) (8). In children, normalized heart mass is 6% greater in males, but after puberty there is a divergence leading to the adult male heart being at least 25% larger (9–12). Postpubertal cardiac growth in males is particularly pronounced in young endurance athletes who continue to train throughout adolescence (13). Sex differences in adult heart mass were initially attributed to differences in lean body mass between men and women, which led to the oversimplification that the female heart was a small version of the male heart. In contrast, the female heart has unique geometry, and its mass does not directly correlate with body mass (14). The male heart is larger in every dimension, but sex differences in cardiac geometry do not scale linearly (14, 15). For example, an echocardiography study of 734 healthy men and women found that while LV mass was 30% greater in men, LV septum and posterior wall thicknesses were just 11% and 9% larger, respectively (16). LV chamber diameter in both sexes scales with heart mass (14). Cardiac mass increases with age in both sexes; however, the sex difference in normalized cardiac mass is diminished in the elderly, among whom the male heart has been reported as being 4%–9% larger on average (17, 18). This is likely due to greater aging-associated LV hypertrophy in females (11, 18, 19). In adults, total blood volume is lower in women, even when normalized to body size (20). It is therefore unsurprising that cardiac output and stroke volume are also reduced (21, 22). Interestingly, women have higher resting heart rates — due to distinct autonomic nervous system regulation of the sinoatrial node (23) — and some studies report they also have 2%- to 3%-higher baseline LV ejection fractions, which culminates in higher cardiac output in women when normalized to lean body mass (14, 23–26). Sex differences also exist in cardiac electrophysiology; however, this topic is outside the scope of the present Review and has been extensively reviewed previously (27).

### Cellular and molecular sex differences.

**Figure 1:**
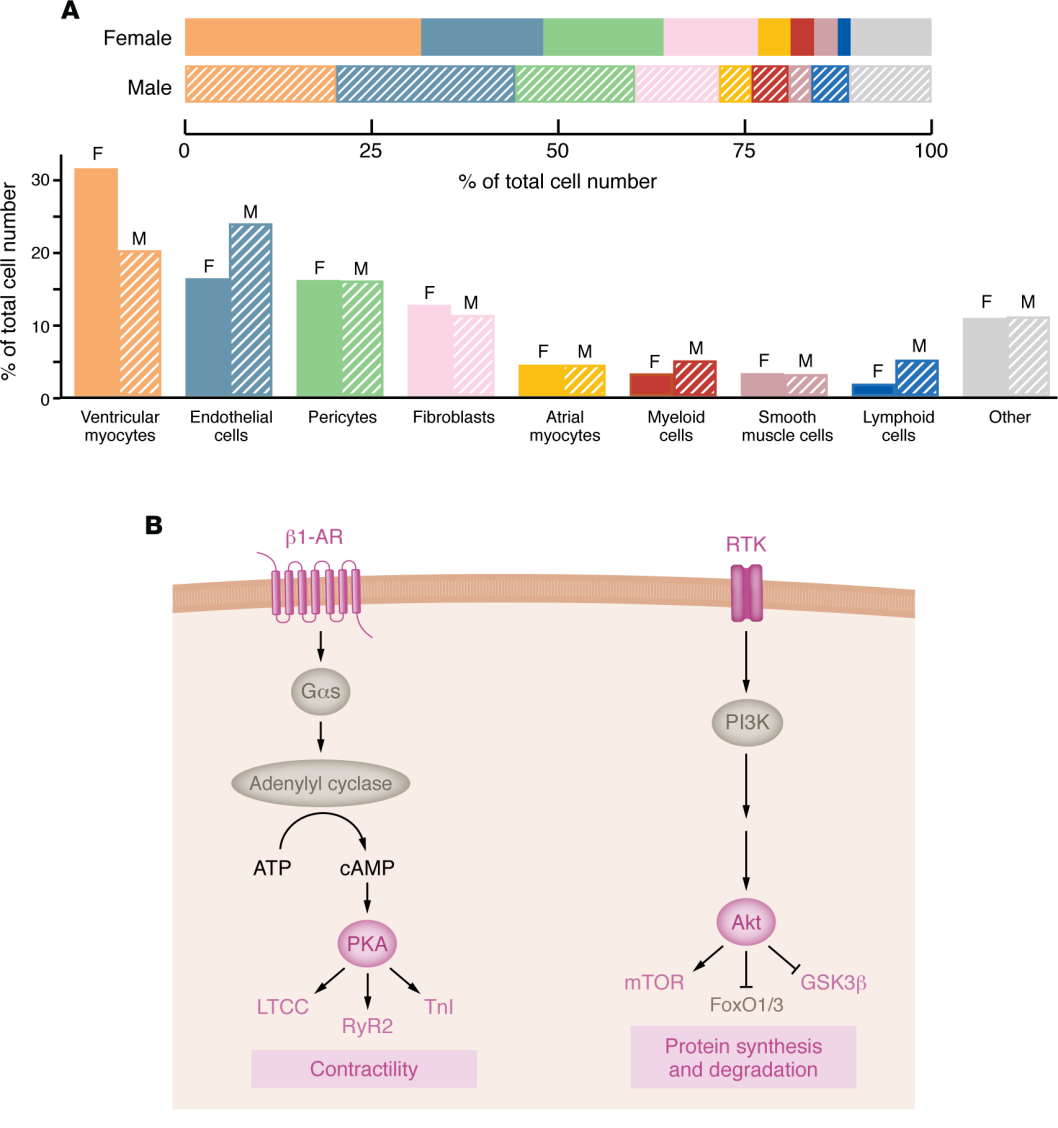
Baseline cellular sex differences in the heart. (**A**) Sex differences in cardiac cell type composition. The adult human heart is approximately 80% cardiomyocytes by mass, but also contains a plethora of other cell types, including fibroblasts, endothelial cells, and immune cells. Sex differences exist most notably in the relative proportion of cardiomyocytes, endothelial cells, and lymphoid cells. Proportions are based on data from single-cell RNA sequencing study performed by Litviňuková et al. (28). Cell types are ordered according to decreasing relative proportions in the female heart from left to right. Adapted with permission from Walker et al. (29). (**B**) Enriched cardiomyocyte signaling pathways in female hearts. PKA and Akt activity are enhanced in females at baseline. Proteins in pink have been directly shown to be enhanced in female hearts at baseline; proteins in gray are implicated based on the pathways activated. PKA phosphorylates proteins involved in contractility, while Akt regulates protein homeostasis. β1-AR, β_1_-adrenergic receptor; Gαs, Gα stimulatory protein; LTCC, L-type calcium channel; RyR2, ryanodine receptor 2; TnI, troponin I; RTK, receptor tyrosine kinase; FoxO, forkhead box protein O.

Cardiac cell type composition differs between sexes (Figure 1A). Male and female humans are born with the same number of cardiomyocytes — the predominant cardiac cell type by mass; however, in adulthood, males have a significantly smaller proportion of cardiomyocytes (28). This is thought to be due to testosterone-induced apoptosis in the male heart (29, 30), although differences in cardiomyocyte-regenerative capacity may also contribute (31). Not all cellular differences can be ascribed to hormones, however, as sex-biases exist prior to gonad development. In mice, male fetal hearts favor cardiac conduction and muscle cell action potential gene ontologies, while female fetal hearts are enriched with genes involved in RNA metabolism (32). In a study of age-matched adult mice, 223 genes and 95 proteins were differentially expressed (32). Examination of healthy human hearts revealed 178 genes that were significantly differentially expressed between sexes (33). In both studies, female hearts were characterized as having increased expression of immune factors. When cardiomyocytes were enriched prior to sequencing, 611 genes were found to be differentially expressed, with female cells displaying increased expression of factors involved in hormone receptor activation and PKA signaling (34). PKA acts downstream of β-adrenergic stimulation and modifies cardiomyocyte excitation-contraction coupling, in which sex differences have been identified in calcium signaling and myofilament function (Figure 1B) (35–37). Sex differences have also been observed for fatty acid metabolism and Akt signaling (Figure 1B) (38, 39). Cellular sex differences contribute to the general resistance to heart disease development in premenopausal women, who display less fibrosis, apoptosis, maladaptive hypertrophy, and inflammation (40–42). These favorable features are generally attributed to estrogen-dependent signaling, since cardioprotection is lost following menopause. The effect of changing hormone levels on cardiac remodeling throughout life is discussed in the following section.

## Contribution of hormones and chromosomes to cardiac sex differences

### Steroid sex hormones.

**Figure 2:**
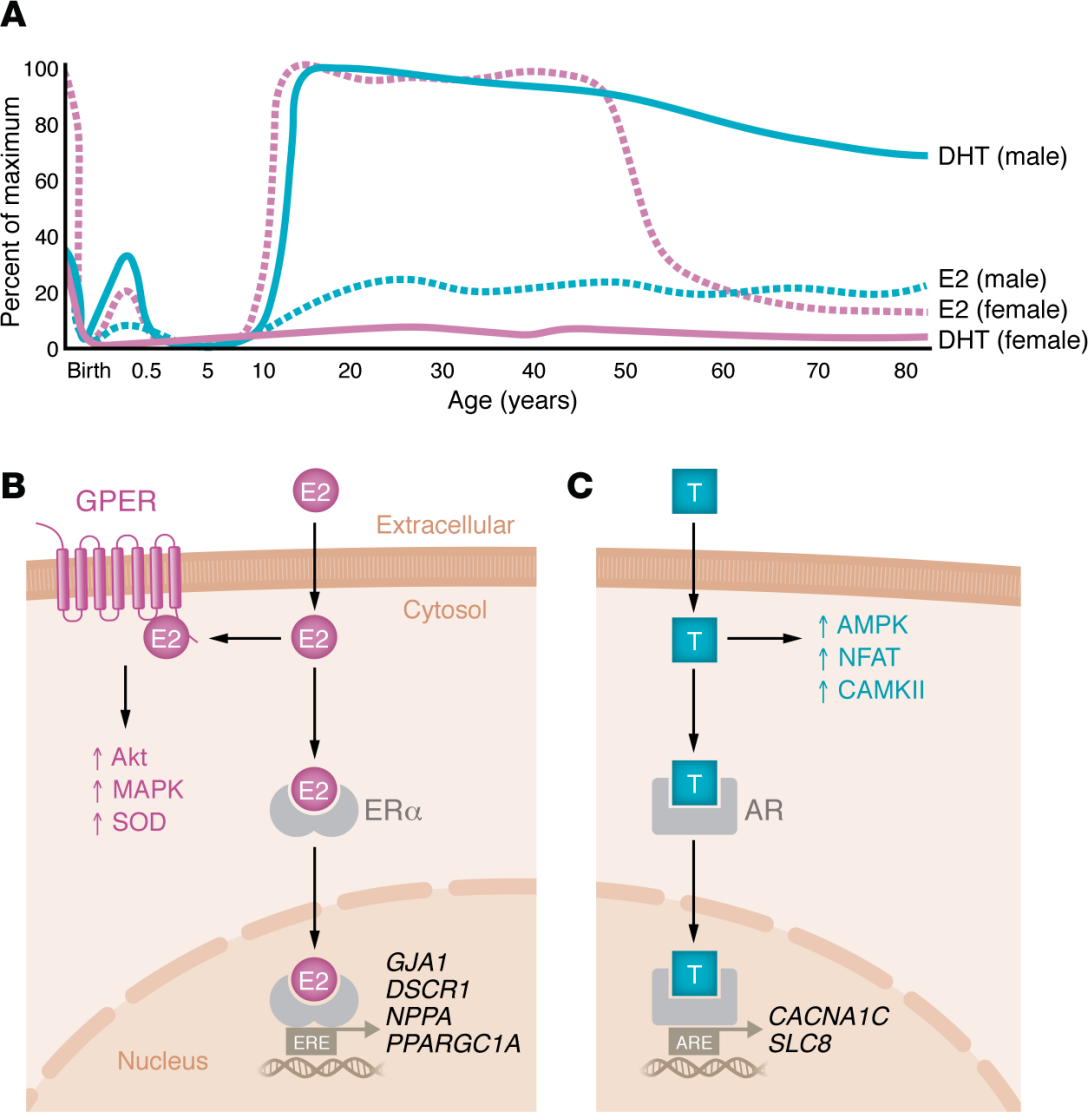
Circulating sex hormones throughout life and their effect on cellular signaling. (**A**) Circulating estrogen (7β-estradiol [E2]) and testosterone (DHT) throughout life in male and female humans. Data are based on those Ober et al. (180) and are represented here with permission from the publisher and copyright holder. (**B**) E2 passively crosses the cardiomyocyte cell membrane and can interact with two different receptors: the G protein–coupled estrogen receptor (GPER) and estrogen receptor α (ERα). GPER signaling likely mediates the nongenomic functions, such as activation of Akt, MAPK, and SOD. ERα translocates to the nucleus and binds to estrogen response elements (ERE) in transcriptional promoters. ERα can also regulate transcription indirectly through interactions with other transcription factors. (**C**) Dihydroxy-testosterone (T) passively crosses cell membranes and interacts with the androgen receptor (AR) in the cytosol. The activated AR displays both genomic and nongenomic functions, which are associated with increased gene expression of calcium-handling genes and activation of AMPK, nuclear factor of activated T cells (NFAT), and calcium-calmodulin-dependent protein kinase II (CaMKII). ARE, androgen response element.

The primary circulating form of estrogen is 17β-estradiol (E2). Both male and female children have circulating E2 concentrations of 5–15 pg/mL, which is secreted by extragonadal tissues (43). However, following menarche, the ovaries become the primary source of E2 in females, who then exhibit circulating levels ranging from 100 to 800 pg/mL depending on menstrual cycle stage (44). Circulating levels drop precipitously following menopause to levels matching those in men of similar ages (Figure 2A) (45). Higher circulating E2 concentrations are inversely correlated with heart disease incidence, and earlier age at menopause increases heart failure risk (46), indicating that E2 contributes to cardioprotection. Notably, women who experienced menopause earlier were also found to have accelerated postmenopausal LV remodeling, including a significant reduction in LV end-diastolic volume (47). Hormone replacement therapy did not mitigate remodeling, suggesting the structural phenotype is not solely the result of decreased cumulative E2 exposure (47). Nevertheless, postmenopausal women with higher circulating E2 levels have reduced disease risk (30). Menopause is also associated with increased myocardial fibrosis, ventricular stiffening, and diastolic dysfunction (48, 49). The receptiveness of the heart to circulating estrogen may also decline with age, as clinical and preclinical studies of E2 replacement in aging females found mixed results and highlight the importance of timing (30, 50). A study in rats found that the antihypertrophic effects of E2 are lost with aging (51), and the cellular effects of E2 in a mouse model of perimenopause were profoundly different from those in reproductively intact animals (52). Thus, it is not clear whether the mechanism of declining cardioprotection in aging females is due to reduced circulating E2, reduced cellular responsiveness to E2, or both. Progesterone is another hormone enriched in females that is known to contribute to cardiac remodeling during pregnancy through activation of calcineurin and protein synthesis (53, 54). However, the effect of progesterone on cardiac remodeling in other settings remains poorly understood.

Androgens, including testosterone, are present in both male and female humans (Figure 2A). Circulating dihydroxytestosterone (DHT) concentrations are similar in male and female children and increase from age 6 through the teenage years (55). However, the increase is much more pronounced in males, who display divergence from females beginning around age 11 with the onset of testosterone production by Leydig cells (55). In most men, circulating DHT levels begin to decline after age 40, but — unlike the rapid reduction in circulating E2 in postmenopausal women — DHT levels fall gradually (Figure 2A) (56). Clinical studies have shown that both high and low testosterone levels are associated with increased risk of developing heart disease (57). Mechanistic studies in mice found reduced cardiomyocyte size and diastolic dysfunction with testosterone deficiency (58), supporting the conclusion that testosterone plays important roles for cardiac structure and function. However, in disease contexts, testosterone is associated with maladaptive hypertrophy and fibrotic remodeling of the LV, which leads to heart failure (59, 60). Compared with that of estrogen, the structural and functional effect of testosterone is relatively less understood and represents an important area for future research.

Cellular signaling initiated by DHT and E2 is mediated by cytosolic androgen receptors (ARs) and estrogen receptors (ERα, ERβ), which have both genomic and nongenomic targets. Genomic functions are mediated by receptor binding to DNA hormone response elements or indirectly through interactions with other transcription factors (61). E2 can also bind to the G protein–coupled estrogen receptor (GPER), a membrane receptor with strictly nongenomic functions (62). In cardiomyocytes, E2 regulates gene expression of the gap junction protein connexin-43 (*GJA1*); metabolic regulatory protein PGC1-α (*PPARGC1A*); atrial natriuretic factor (*NPPA*); and MCIP1 (*DSCR1*), a negative regulator of calcineurin (61). Notably, only ERα is expressed in rat cardiomyocytes, suggesting that the protective benefits of ERβ in cardiac remodeling stem from its effect on other cardiac cell types (63). Established nongenomic actions of E2 include modulation of PI3K/Akt, MAPKs, and the cellular antioxidant response through superoxide dismutase (SOD) (64). The modulation of PI3K and MAPK signaling likely arises through GPER-dependent regulation, as ERα is not competent to activate these pathways (63). AR-mediated cell signaling is not well understood; however, DHT exposure is known to activate pathology-associated factors in cardiomyocytes, including CaMKII and nuclear factor of activated T cells (NFAT) (65). ARs also directly regulate gene expression of the L-type calcium channel (*CACNA1C*) and sodium-calcium exchanger (*SLC8*), which contributes to alterations in calcium signaling between sexes (66). The effects of E2 and DHT on cardiomyocyte biology are summarized in Figure 2B.

### Sex chromosomes.

Hormones play important roles in growth and remodeling; however, the heart develops prior to the gonads, and sex differences already exist at these early time points, suggesting involvement of sex chromosomes. Compared with that of hormones, the role of sex chromosomes in cardiac remodeling is poorly understood. The X and Y chromosomes contain approximately 800 and 78 protein-coding genes, respectively, and their expression in nongonadal tissues contributes to sex differences (67). The X chromosome also contains 118 miRNAs, which represents approximately 10% of the total known miRNAs (68). The involvement of miRNAs in cardiac remodeling is well established and has been reviewed previously (69). To account for X chromosome dosage differences, female somatic cells employ X inactivation — a mechanism whereby one X chromosome is functionally silenced by the RNA *XIST* (70). However, 15%–25% of X chromosome genes escape inactivation in humans, leading to increased relative expression of these genes in women (71, 72). X-linked genes account for approximately 30% of differentially expressed proteins between male and female mouse hearts at baseline (32). In mice, having two X chromosomes is associated with worse remodeling following ischemia/reperfusion injury, irrespective of hormone status (73). This coincides with increased expression of genes that escape X inactivation, including the elongation initiation factor *Eif2s3x* and lysine methyltransferases *Kdm6a* and *Kdm5c* (73). Genes that escape X inactivation have also been linked to activation of aortic valve myofibroblasts, which may underly sex differences observed in AS (discussed below) (74). Genes that escape X inactivation are tissue dependent, differ between species, and remain to be comprehensively investigated in cardiomyocytes. The role of X and Y chromosome genes in cardiac remodeling is largely unknown and represents an important future direction for research into cardiac sex differences.

## Sex differences in physiological cardiac remodeling

### Human studies.

**Figure 3:**
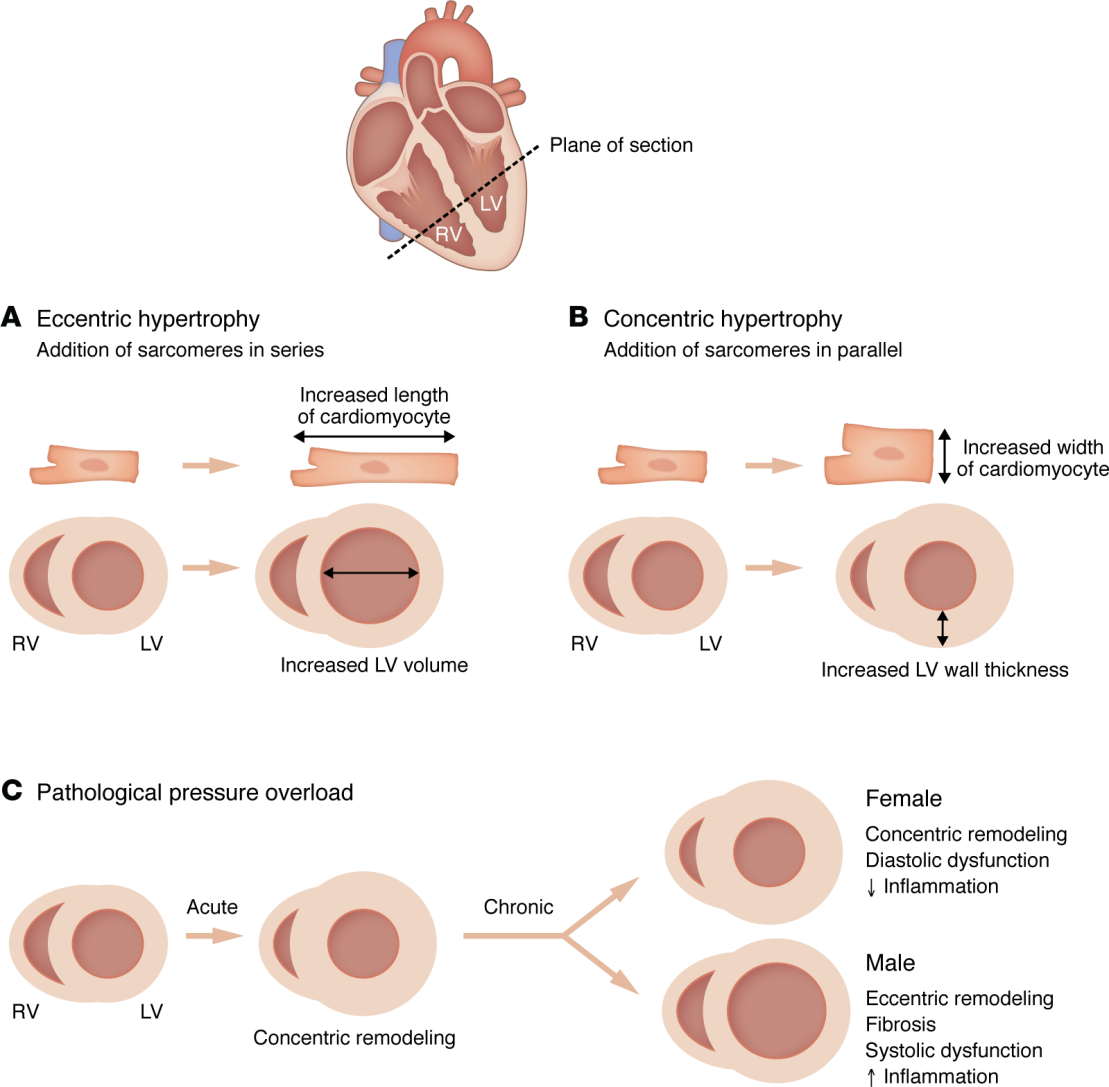
Cell- and organ-level remodeling in eccentric and concentric hypertrophy. (**A**) Eccentric remodeling in cardiomyocytes is associated with increased cardiomyocyte length-to-width ratio, stemming from the serial addition of contractile units known as sarcomeres. At the organ level, this manifests as increased LV chamber diameter. Eccentric cardiac hypertrophy is most common in conditions of volume overload, such as with endurance exercise training or aortic regurgitation. (**B**) Concentric remodeling arises from the addition of sarcomeres in parallel, which reduces the cardiomyocyte length-to-width ratio. The result is increased LV septum and free wall thickness and, in healthy conditions, this does not coincide with reduction in LV chamber volume. In pathological settings of pressure overload, such as AS or chronic hypertension, concentric remodeling is associated with reduced LV chamber diameter. (**C**) Sex differences in cardiac remodeling with pathological pressure overload.

Volume overload, which increases LV preload, is the primary stimulus for adaptive cardiac growth with endurance exercise (75). Blood volume increases by 20%–25% with endurance training in both sexes (76), and, to reduce wall strain, the LV compensates by increasing chamber diameter and wall thickness through eccentric cardiomyocyte hypertrophy (Figure 3A) (7). Adaptive physiological cardiac growth is most common in rowers, but also occurs frequently in runners, swimmers, cross-country skiers, and cyclists (77). This hypertrophy develops rapidly and is associated with improved cardiac function (7). Across sporting disciplines, female athletes display smaller indexed LV mass and wall thickness, and these differences are sustained when normalized by training volume (Figure 3C) (78–82). Sex differences in LV chamber diameter are less pronounced, with some studies reporting no difference or even larger relative chamber dimensions in women (78–81, 83, 84). This suggests that structural remodeling in the female athlete’s heart especially favors increasing chamber diameter over wall thickness, which is supported by studies showing that female endurance athletes almost exclusively develop eccentric hypertrophy (78, 79). Exercise-induced LV remodeling in both sexes favors eccentric remodeling, although a study of 947 elite athletes (22% female) found that LV wall thicknesses in some men (2.2%) entered the diagnostic range for hypertrophic cardiomyopathy (77). Another study, of 1,083 athletes (41% female), found that 4% of highly-trained female athletes experienced concentric hypertrophy (Figure 3B), compared with 15% of men (78). These collective studies support the conclusion that the type of LV remodeling that occurs with exercise is influenced by biological sex. Women also display unique biventricular adaptations to exercise, with higher LV/RV mass ratio compared with men (79). Unlike most instances of pathological hypertrophy, exercise-induced LV remodeling is rapidly reversible (7). Detraining in athletes for 40–240 days following the 1988 Olympic Games caused an average 31% reduction in LV wall thickness (77); and an acute, 32% increase in LV mass that occurred during a 6-day, 622-kilometer ultramarathon was completely reversed 3 days after the race (85). Sex differences in regression have been described for both physiological and pathological hypertrophy; however, regulation of reverse remodeling is outside the scope of this Review and has recently been reviewed elsewhere (7).

### Rodent models.

**Figure 4:**
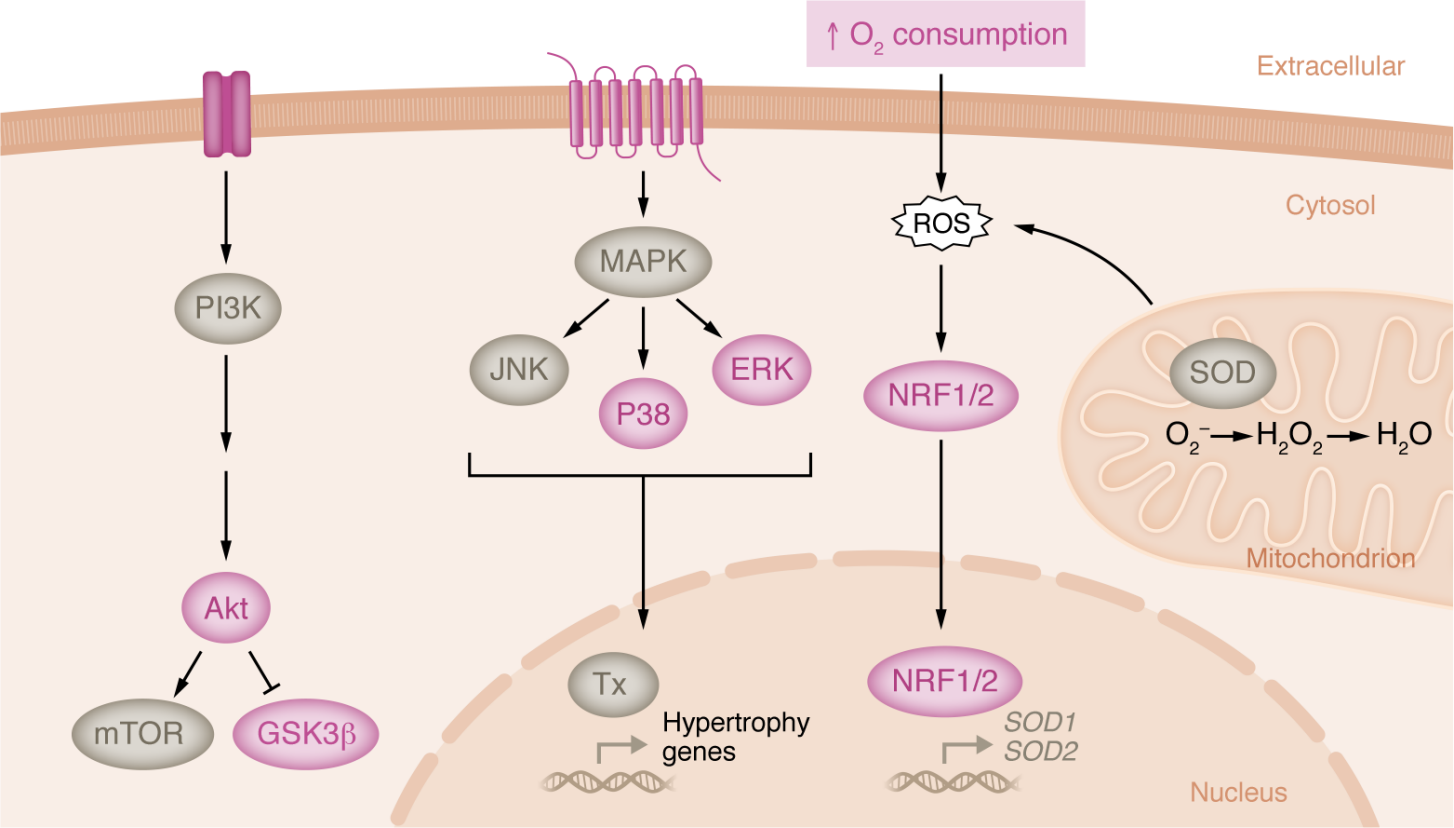
Cellular signaling associated with exercise-induced cardiac hypertrophy in female mice. Cardiac hypertrophy with exercise is pronounced in female mice compared with males. At the cellular level, female mice display increased Akt activation, which regulates protein synthesis through modulation of mTOR and GSK3β. Female hearts also display activation of factors in MAPK signaling cascade, including P38 and ERK, which indirectly control transcription of a hypertrophic gene program via direct modulation of various transcription factors (Tx). The female mouse heart also has increased expression of nuclear factor erythroid 2–related factors 1 and 2 (NRF1 and NRF2) after exercise. NRFs translocate to the nucleus in response to oxidative stress from ROS and upregulate expression of antioxidant factors, including SOD1 and SOD2. Factors in pink have been directly shown to be induced with exercise; factors in gray are implicated based on the pathways activated.

Opposite to what occurs in humans, female mice display greater exercise-induced hypertrophy than males, irrespective of exercise type or training volume (86–89). Swimming induces more remodeling than running but is also associated with an increase in stress-induced catecholamine release (90, 91). Voluntary wheel running, which does not cause stress, is therefore a more appropriate model; it induces a 10%–25% LV mass increase that peaks after 2–4 weeks of training (86, 92, 93). Hypertrophy is influenced by genetic background, as FVB/NJ mice display a more potent sex difference than C57BL/6J mice (86). Female mice run more daily distance than males, yet hypertrophy normalized to distance run remains higher than in males (86, 88, 92, 94). Exercise also increases plasma free fatty acid and triglyceride levels to a greater extent in females (89), suggesting that circulating factors may contribute to hypertrophy development as in other species (95). At the molecular level, trophic signaling pathway activation is enriched in females. Female mice display a higher proportional increase in CaMKII activity and sustained suppression of GSK3β with exercise (86). Activation of Akt has been observed in both sexes but is enhanced in females (86, 89), while AMPK activation is unique to males (92). MAPK signaling is also activated in females, which have increased phosphorylation of ERK and P38 (Figure 4) (94). The female heart is also enriched with the antioxidant factors NRF1 and NRF2 following exercise (Figure 4) (94). It is not known whether these same factors are induced with exercise in the human female heart; however, studies of human skeletal muscle adaptation with exercise identified sex differences in fatty acid metabolism, mitochondrial biogenesis, and Akt signaling, all of which were increased in women (96, 97). The sex difference in exercise-induced hypertrophy in mice is dependent on estrogen receptor β (ERβ) signaling, as ERβ-knockout females display reduced hypertrophy with the same running volume (94). ERβ knockout also prevents the exercise-induced activation of Akt, MAPKs, and protein synthesis in females (94). Notably, estrogens appear to have a suppressive effect on hypertrophy in males, as switching mice to a casein-based diet from traditional soy-based mouse chow, which is rich in phytoestrogens, resulted in greater hypertrophy (92). Female mice displayed equivalent hypertrophy with soy and casein (92). In adult men, high circulating estrogen levels are associated with increased myocardial fibrosis (98). The high phytoestrogen content in traditional mouse chow likely plays a major role in the observed discrepancy between the human and mouse data in exercise-induced cardiac hypertrophy between males and females.

## Sex differences in pathological cardiac remodeling

### Pressure overload.

AS is a calcific valve disease that increases LV afterload and is present in more than 5% of the population over 65 years of age (99, 100). The frequency of AS is similar between males and females, although the disease is likely underdiagnosed in women (100, 101). AS progresses at a similar rate in both sexes (102); however, women maintain better systolic and diastolic function, regardless of AS severity (102–109). All-cause mortality with AS has been reported as not being different between sexes, although some studies found reduced mortality in women (100, 102, 110). The sex difference in systolic function stems from maintenance of higher peak LV pressures in women, which is due to differing LV geometry (106, 107, 109). LV mass index is typically lower in women, and they develop a more concentric form of hypertrophy, while maladaptive LV dilation is more prevalent in men with chronic pressure overload (Figure 3C) (103, 104, 108, 111). This leads to female hearts having greater relative LV wall thickness (107, 109). Male hearts have higher extracellular volume, suggestive of more fibrosis (106), which is supported by the finding that LVs from male AS patients have higher collagen gene expression (111). Female sex is associated with suppression of extracellular matrix (ECM) and inflammatory gene expression (111). AS severity in men directly correlates with circulating natriuretic factor levels, whereas these biomarkers do not track with disease severity in women (106, 108).

Preclinical studies of pressure overload use the transverse aortic constriction (TAC) rodent model (112). Cardiac hypertrophy and induction of pathological molecular factors are observable within 1 week after TAC (113). Most studies show that the extent of hypertrophy 2–4 weeks after TAC is similar in males and females (114–116); however, males develop greater LV hypertrophy with longer-term pressure overload that is associated with increased fibrosis (115, 117, 118). As in humans, female rodents develop a more concentric form of hypertrophy and maintain higher peak LV pressures, while males transition into heart failure earlier, experience greater systolic dysfunction, and display LV dilation (114, 118). Reduced systolic function in males arises from suppression of contractile reserve (116). At the cellular level, dysfunction is linked to reduced sarcoplasmic reticulum calcium ATPase (SERCA2A) and increased β-myosin heavy chain (MYH7) expression (116). Male hearts also have increased expression of ECM genes, suppression of mitochondrial antioxidant factors, and increased apoptosis after TAC (115, 117–120). Fibrotic remodeling in males stems from androgen-dependent TGF-β activation, and inhibiting this pathway through TGF-β neutralization or gonadectomy prevents fibrosis (119). Sex differences with regard to pressure overload are also linked to ERβ signaling. Mouse studies found that protection against fibrosis and apoptosis in females was abolished with ERβ knockout, which also led to greater hypertrophy in females after TAC (117, 118, 121). Mechanistically, ERβ helps maintain cardiac function after TAC through inhibition of inflammatory pathways and maintenance of mitochondrial metabolism (117, 120). ERα ablation has no effect on hypertrophy development with pressure overload (121). Together, the human and rodent data support the conclusion that estrogen is protective against maladaptive pressure overload–induced remodeling through modulation of ECM deposition and cardiac inflammation, while testosterone enhances pathology in disease contexts.

### Volume overload.

Pathological cardiac remodeling can also arise in conditions of chronic volume overload — although this is a less-common cause than pressure overload — as occurs with aortic regurgitation and mitral regurgitation. Volume overload induces eccentric remodeling due to the increased pressure against the LV walls resulting from higher chamber volume. Sex differences in responses to chronic volume overload are consistent with those observed for pressure overload, and females generally experience less adverse remodeling. Clinical studies found reduced LV volume, reduced LV mass, and increased LV ejection fraction in women compared with men across the spectrum of aortic regurgitation severity (122–126). However, the female heart exhibits greater expansion of extracellular volume relative to the male heart, suggesting an association between chronic volume overload and higher levels of fibrosis (122). A study of skinned cardiac fibers from male and female patients with mitral regurgitation found that female fibers had higher developed force at maximum calcium concentrations, indicating that sex differences in the response to volume overload extend to the myofilament level (127). In a rat model of volume overload, hearts from males displayed increased LV hypertrophy and chamber dilation compared with female hearts (128). Males also had 10-fold-higher mortality, further supporting that females are protected against pathology involving volume overload (128). In mice with aortic regurgitation, LV remodeling in males was associated with activation of CaMKII and Akt, increased fetal gene expression, and induction of apoptosis factors, including Bax and cleaved caspase-3 (129). Another study showed that volume overload from atrioventricular shunt caused apoptosis and increased Bax and caspase-3 and -9 expression in the male rat heart, while hearts from females did not exhibit increased apoptosis, which was estrogen dependent (41). Despite evidence of reduced adverse remodeling in the female heart in response to pathological volume overload, women are more likely to experience symptoms with aortic regurgitation, are older at the time of diagnosis, and have worse prognosis (122, 124, 125), suggesting that the condition may be underdiagnosed.

### Ischemia.

Coronary artery disease (CAD) contributes to increased risk of myocardial infarction (MI), the number one cause of death and morbidity in the United States, and manifests differently in men and women (130, 131). MI occurs a decade earlier on average in men due to earlier development of CAD (132); however, despite maintaining higher LV ejection fractions and stroke volumes than men after MI (131), women have an increased risk of developing heart failure and display higher mortality, especially at younger ages (133–136). Adverse remodeling occurs in half of all patients after MI, irrespective of sex, and is denoted by LV chamber dilation, wall thinning, and systolic dysfunction (137). However, men display significantly larger normalized LV chamber size and mass (134, 138, 139). Differences in remodeling are reflected at the cellular level, where myocyte volume and length are also greater in males (138). Transcriptional responses to MI also differ, with one study identifying 271 differentially expressed genes between sexes in LV biopsy samples from patients with ischemic heart disease (140). Pathway analysis of these genes identified oxytocin- and estrogen-dependent signaling as the top two significantly enriched pathways in the female heart (140).

In rodent models of MI from coronary artery ligation, female sex is associated with protection against adverse structural and functional outcomes (59, 141–143). Males have higher rates of cardiac rupture and neutrophil infiltration after MI, while female hearts display greater recruitment of macrophages and reparative monocytes (142, 144, 145). The increased proinflammatory response and activation of MMPs in males contribute to scar thinning (144, 146); however, one study found that MMP inhibition reduced rupture incidence by half (146). Both male and female mouse hearts display increased activation of MAPK signaling, denoted by phosphorylation of P38 and ERK1/2, while females additionally show activation of STAT3, which is estrogen dependent (147). In ovariectomized females, estrogen replacement is associated with worse outcomes in the acute phase after MI but better LV structure and function with chronic exposure (148), which may explain the increased risk of mortality in young women. Estrogen-dependent signaling is linked to improved long-term remodeling and reduced apoptosis and inflammation (144). These benefits are conferred by ERβ, and knockout of the receptor ablates protection from ischemic heart disease in female mice (143). Meanwhile, testosterone causes greater hypertrophy development and increased cardiac rupture risk in males (59, 142, 149). Interestingly, E2 administration in male mice suppresses the post-MI decline in systolic function and mitigates some of the adverse LV remodeling (59, 144). E2 administration in males is also associated with a reduction in proinflammatory cytokines and suppression of P38-dependent apoptosis (144). These collective findings support the conclusion that testosterone-dependent signaling in the heart worsens pathology in the context of ischemia, while the effects of estrogen are timing dependent.

## Sex differences in remodeling with genetic cardiomyopathies

Genetic heart disease is estimated to account for approximately 25%–30% of all heart failure cases (150) and is broadly categorized into four subclassifications: hypertrophic cardiomyopathy (HCM), dilated cardiomyopathy (DCM), restrictive cardiomyopathy (RCM), and arrhythmogenic right ventricular cardiomyopathy (ARVC). Biological sex can affect disease penetrance, onset, and pathogenesis in each of these conditions (151). In this section, we discuss the leading monogenic causes of HCM and DCM, the most common genetic heart diseases, and highlight the sex differences in disease development and structural remodeling.

### HCM.

HCM is the most common form of inherited heart disease, occurring at a frequency of 1:250 to 1:500, and is also the leading cause of sudden death in adolescence (152). It is defined morphologically as LV hypertrophy in the absence of abnormal loading conditions, such as AS or hypertension (153). Missense and truncating mutations, respectively, in the genes encoding β-myosin heavy chain (*MYH7*) and cardiac myosin binding protein C (*MYBPC3*) are the most common causes of HCM (152). HCM has an autosomal dominant inheritance pattern and so is expected to occur at equal rates in men and women (152); however, women are more likely to present with pathogenic sarcomere variants (51% vs. 43% for men) (154). Disease onset in women occurs 3–7 years later on average, although they typically present with more-advanced disease and experience worse outcomes (154–157). Disease onset with *MYH7* mutations is similar in the two sexes but is delayed in women with *MYBPC3* mutations (154, 158, 159). HCM disease penetrance is typically higher in men, and the disease is found more commonly in men until age 60 years, suggesting that premenopausal women are partially protected (158, 160). Women with HCM have smaller LV diameters and increased relative septal and posterior wall thicknesses, and experience more LV outflow tract obstruction and diastolic dysfunction (154, 155, 161, 162). Adverse remodeling is also more prevalent at the cellular level in the female heart, which displays more fibrosis and lower capillary density than the male heart (161). Female sex is also associated with increased mortality, regardless of age or comorbidities (155). In mice, a missense mutation in myosin heavy chain (R403Q) — the first causative mutation identified for HCM — recapitulates human disease phenotypes (163). Studies of this model suggest that disease onset and progression are dependent on biological sex. At 3–4 months, males and females exhibit a similar degree of hypertrophy (164); however, 10-month-old females display concentric hypertrophy, while males progress into LV dilation with systolic dysfunction at this time point (163–165). These data support the conclusion that females are partially protected, which aligns with older age of diagnosis in female patients.

### Dilated cardiomyopathy.

DCM is characterized by LV dilation and reduced systolic function (166). Unlike that occurring with endurance training, chamber dilation with DCM is associated with LV wall thinning. Approximately 40% of all DCM cases have a known monogenic cause (151). DCM is nearly as prevalent as HCM and is characterized by autosomal dominant inheritance for all but one known gene: *DMD*, encoded by the X chromosome (166, 167). Sex biases are not observed with pediatric DCM; however, in adults, women account for just 30% of total cases (151). Women with DCM maintain higher LV contractility, exhibit less dilation, and have reduced fibrosis (168). Despite these favorable characteristics, adverse outcome risk is higher in women (168). The leading genetic causes of DCM are truncating mutations in the gene encoding the sarcomeric protein titin (*TTN*) and missense mutations in lamin A (*LMNA*), a component of the nuclear membrane (169). Women with DCM from *TTN* or *LMNA* mutations typically fare better than men with respect to clinical outcomes (170, 171). Titin-truncating variants are the most common cause of DCM, accounting for approximately 20% of all cases (172, 173). In these patients, DCM typically presents in midlife and occurs earlier in men (170, 174). Titin mutations show less disease penetrance in women, who are also less likely to experience adverse remodeling and more often experience reverse remodeling with guideline-directed medical therapies (7, 170, 172, 174). Risk of ventricular arrhythmias is also lower in women with DCM due to *TTN* mutations (174). *LMNA* mutations account for approximately 5% of all DCM cases, and disease penetrance in adulthood is over 90% (151, 169). Men with *LMNA* mutations have increased risk of ventricular arrhythmias, heart failure, and mortality (171, 175). They also more frequently develop systolic dysfunction (175). In mouse models of genetic DCM, disease onset is earlier in males, which exhibit enhanced cardiac remodeling and disease progression that is linked to androgen signaling (176).

These collective studies suggest that remodeling develops differently in men and women with genetic heart diseases. Women also tend to have delayed disease onset but experience worse outcomes, which may be partially due to lesser awareness of the disease in women and/or to disease diagnosis occurring at later stages.

## Important future directions

### Modeling cardiac sex differences in in vitro systems.

Thus far, mechanistic investigations into cardiac sex differences have employed rodent models. These models, while valuable, fail to replicate human sex differences in cardiac remodeling in some cases. Moreover, while over 15% of human X chromosome–encoded genes escape inactivation, only 3% escape in mice (177). Thus, there is a need for new models to investigate mechanistic sex differences in humans. Human induced pluripotent stem cell–derived cardiomyocytes (iPSC-CMs) could represent such a model and have the added benefit of greater throughput compared with animal models; however, they are limited by their immaturity and failure to replicate metabolic and functional features of adult cardiomyocytes. Culturing iPSC-CMs in three-dimensional systems (e.g., engineered heart tissues [EHTs]) with fatty acid–rich maturation media improves cell maturity and enables replication of many features of adult cardiomyocytes (178). To our knowledge, there have been no studies investigating cellular sex differences in iPSC-CMs with or without the addition of relevant hormones. Future studies using EHTs should investigate whether these platforms recapitulate human sex differences in cardiomyocytes differentiated from male and female iPSC lines with physiological concentrations of sex hormones. Additionally, the role of X inactivation in iPSC-CMs should be investigated.

### Consideration of biological sex with disease diagnosis and treatment.

One takeaway from clinical studies is that there is a clear need for sex-specific diagnostic criteria in both structural and functional characterizations of heart disease to avoid underestimation of disease in women (151, 179). Relative LV wall thickness in the male heart is greater at baseline, so reaching the diagnostic range for pathological cardiac hypertrophy (≥13 mm) requires less remodeling in men compared with women. Thus, if traditional diagnostic guidelines are followed, the degree of remodeling at diagnosis is expected to be substantially greater for women. This leads to risk of disease diagnosis at later heart failure stages in women, which may contribute to their observed greater heart disease mortality. Sex-specific criteria for cardiac dysfunction may also be valuable, since higher baseline LV ejection fractions have been reported in women (25). Increasing awareness around the high rates of heart disease mortality in women, and how the disease often manifests differently than in men, will surely bring additional benefits with respect to clinical outcomes.

## Summary

There are profound sex differences in cardiac remodeling in response to physiological and pathological stimuli. These are borne out at the cellular level through biased cellular signaling mediated by sex hormones and chromosomes, which leads to the premenopausal female heart being partially protected against heart disease. Cardiac remodeling in all contexts may be further modified by age, genetic background, and diet, which can act to enhance or mitigate sex differences. Future studies should emphasize further mechanistic investigations into cardiac sex differences, particularly the role of sex chromosomes, which has been relatively understudied to date. Clarifying these sex-specific mechanisms of remodeling is essential to inform future therapeutic development for the benefit of both men and women with heart disease.

## Version 1 07/01/2024

Electronic publication
